# Participation in the digital transformation of healthcare: a review of qualitative studies

**DOI:** 10.1108/IJHCQA-03-2024-0021

**Published:** 2024-11-15

**Authors:** Lisabet Wieslander, Ingela Bäckström, Marie Häggström

**Affiliations:** Department of Quality Management, Mid Sweden University, Sundsvall, Sweden; Vård och Omsorgsförvaltningen, Sundsvall, Sweden; Department of Quality Management and Mechanical Engineering, Mid Sweden University Campus Ostersund, Ostersund, Sweden; Department of IHV, Mid Sweden University, Sundsvall, Sweden

**Keywords:** Health professionals, Involvement, Participation, Quality improvement, Change management, Organizational improvement

## Abstract

**Purpose:**

The purpose of this review is to identify how health professionals perceive participation in implementation of new technology in healthcare organizations.

**Design/methodology/approach:**

A qualitative systematic review based on the PRISMA diagram, was conducted using qualitative synthesis. NVivo software was used for thematic analysis. The searches were performed in PubMed, CINAHL and Scopus.

**Findings:**

A total of 15 articles were included in the review, four themes describing how participation of health professionals in digital transformation affects the outcomes were identified, and three themes describing the factors that are necessary to promote participation. The underlying latent theme of an unmet desire to participate in the digital transformation was also identified in the analysis.

**Originality/value:**

The digital transformation of healthcare is complex and faces many obstacles if not managed correctly. Professional participation in the implementation seems to be essential for success. Focus on increased resources and planning during early stages, as well as teamwork and ethical reflection is important addressing the challenges that professionals face in digital transformation of healthcare.

## Introduction

The implementation of new technology is viewed as a solution to various challenges faced by healthcare organizations; however, this process has been shown to be complex and slow (Hellstrand *et al.*, 2024; Øvretveit, 2017). The contemporary integration of new technology into healthcare is commonly referred to as digital transformation (Øvretveit, 2017; Tomar *et al.*, 2023). These technological advancements may enhance the quality of care, decrease expenses, and address challenges related to workforce management (Herrmann *et al.*, 2018). Digital transformation is understood as a process of organizational redesign through the integration of digital solutions (Mergel *et al.*, 2019; Vial, 2019; Warner and Waeger, 2019). New technologies can include more efficient electronic health record systems and welfare technologies that enhance patients' quality of life. Examples include video equipment for maintaining contact with relatives, reducing feelings of loneliness, or advancements in medical technology that contribute to improving care and reduce risks, such as alarm systems and innovative mattresses designed to prevent pressure ulcers. The essence of digital transformation lies not only in the adoption of digital tools but also in fostering a shift in attitudes, behaviors, and thinking within the organization (Gimpel *et al.*, 2018). While new technologies are consistently emerging, the expected digital transformation has not materialized on a wide scale as anticipated (Hwang and Christensen, 2008; Perakslis, 2017). Additional research on the digital transformation of healthcare is essential for obtaining insights into the factors that delay this process as well as identifying the necessary facilitators thar can ease the implementation processes (Persson and Rydenfält, 2021).

In the elder care in Western countries, significant challenges emerge with regard to the recruitment and retention of health professionals (Christensen *et al.*, 2000; Rönnqvist *et al.*, 2015). Moreover, these nations face substantial demographic shifts involving a growing population of elderly who live longer and experience multiple health conditions that increase their care requirements (Barracca, 2015). The convergence of these factors exacerbates existing social problems (European Commission, 2018). Addressing this challenge is imperative regarding the attainment of high-quality care for elderly.

Policy-makers and healthcare organizations view digital transformation as a potential solution to the shortage of health professionals, due to their expectation that this can process more time available for the delivery of care (Leung *et al.*, 2021). However, digital transformation represents a complex and relatively novel development within healthcare, and the methods associated with and extent of the implementation of new technologies remain uncertain. Large healthcare organizations often exhibit resistance to change, and health professionals frequently perceive that new technologies are inadequately integrated into daily routines, resulting in increased stress and complications rather than the anticipated relief and time savings (Golz *et al.*, 2021; Lövestam *et al.*, 2020). One study explored nurses' perceptions of implementing new technology, revealing that only half of such processes received positive ratings from nursing staff (Nienke *et al.*, 2011). This situation highlights the necessity of exploring innovative approaches and scrutinizing the processes used to enhance the implementation of new technologies in healthcare.

Healthcare organizations are complex and interactive systems in which multiple factors can influence the implementation of new technology (Booth and Carroll, 2015). To address these issues and ensure that health professionals not only accept but also derive value from the technology being introduced, it is suggested that the involvement of employees in the digital transformation may lead to more contented staff and facilitate more effective technology implementation and utilization (Jones and Smith, 2004; Kostman and Sastry, 2019). Nevertheless, the phenomena underlying employee participation in this context and the measurements required to foster a supportive organizational culture that can promote employee participation in the digital transformation remain uncertain. The objective of this literature review is therefore to investigate qualitative studies that have explored the process of digital transformation, with a particular emphasis on health professionals' perspectives on employee participation.

### Aim

The purpose of this review is to identify how health professionals perceive participation in implementation of new technology in healthcare organizations.

Research questions:

RQ1. What organizational factors are needed for enabling employee participation in the implementation of new technology within healthcare?

RQ2. What does employee participation contribute with to technology implementation?

## Background

In recent years, numerous conceptual frameworks for change, such as Kaizen, Lean Management, and Total Quality Management (TQM), have increasingly emphasized the importance of employee participation (Klein *et al.*, 2020; Zink, 2008). The significance of employee participation in quality improvement initiatives has been demonstrated in previous research (Brumback, 2008). Participating in organizational improvement has been suggested to provide employees with challenges, thereby enriching their jobs, fostering commitment, and reducing turnover rates (Zink, 2008). However, only limited evidence has been found to support this notion in the context of healthcare and digital transformation. Historically, one example of employee participation has taken the form of quality circles in Japan, a strategy that has been associated with widespread success (Munchus, 1983; Zink, 2008). Particularly in light of the rapidly evolving changes that occur in modern organizations which are driven by digital transformation (Odone *et al.*, 2019), employee participation has been described as a crucial success factor for any organization (Carballo, 2023; Klein *et al.*, 2020).

The implementation of new technologies in healthcare introduces changes to the work environment and workflows of health professionals, potentially leading to unintended consequences for patient safety (Poon *et al.*, 2021). While new technologies entail positive changes, such as advancements in healthcare practices, they may also contribute to increased workload (Gesner *et al.*, 2019). Consequently, the adoption of these technologies may have unforeseen and mixed impacts on professionals and care (Lee, 2021; Walker *et al.*, 2020; Wisner *et al.*, 2019). Such impacts not only have implications for patient safety and workforce retention at the organizational level but also influence the overall well-being of health professionals at the individual level (Jedwab *et al.*, 2022; Melnick *et al.*, 2021).

Quality management (QM) and nursing science exhibit similar core values, thus making it possible for these fields to learn from one another and to find joint solutions to complex problems in the context health care (Sten *et al.*, 2020). The theoretical assumptions underlying this review are mainly drawn from the field of QM. The focus of QM research has evolved from defining QM practices to measuring them and exploring the relationship between QM practices and performance (Barata and Cunha, 2017; Flynn *et al.*, 1995; Kaynak, 2003; Pereira-Moliner *et al.*, 2016; Xu *et al.*, 2020). One fundamental principle of QM theories is the need to foster commitment and involvement from everyone in organizational development (Bakotić and Rogošić, 2017; Kaynak, 2003; Waldman, 1994; Xu *et al.*, 2020). This task includes optimizing processes and fostering the active participation of individuals throughout the organization (Deming, 2000; Jurburg *et al.*, 2017).

## Method

This review was conducted as a qualitative systematic review (Sandelowski *et al.*, 2007), and systematic search methods were employed with the PRISMA flow-chart (Elkhoury *et al.*, 2022). The review of qualitative literature enabled the researchers to adopt an inductive approach that allowed all factors affecting the phenomenon of interest to be explored (Dixon-Woods *et al.*, 2006; Rachel de Carvalho *et al.*, 2010; Sandelowski *et al.*, 2007; Whittemore and Knafl, 2005).

The review process started with the identification of the research question based on an initial literature search. According to Gough *et al.* (2012) the qualitative review exhibits certain similarities with grounded theory in that the research question can be formulated and redefined during the process.

The databases used were CINAHL, Scopus and PubMed. Only primary qualitative research articles written in English were included. The search terms used were chosen to intercept as many articles as possible with the aim of understanding employee participation in the digital transformation of healthcare and are presented in Appendix 1.

**Figure 1:**
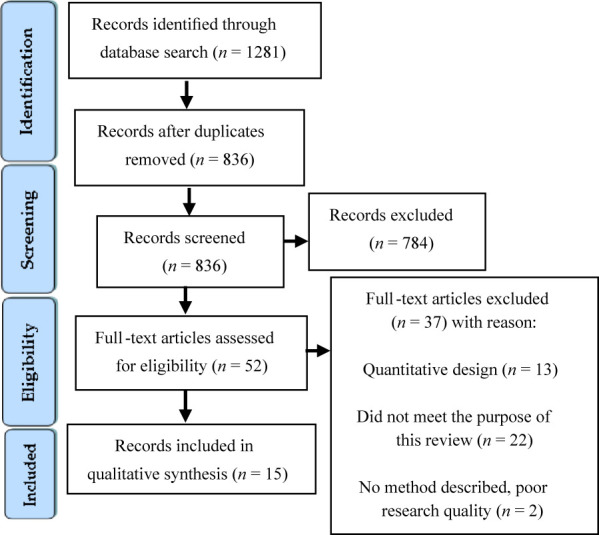
Presentation of the search process–using PRISMA chart flow

A total of 1,281 hits were revealed in the database searches (Figure 1). Of these articles, 15 were selected because they were in line with the aim of this review. This scarcity of articles investigating employee participation in the implementation of new technology in healthcare organizations highlights a research gap. The thorough process of reviewing numerous articles to identify only 15 that were relevant for this review was considered crucial to ensure no important studies were overlooked.

The rigor of the review process was enhanced by several meetings with the review team and by the adoption of a review framework (Braun and Clarke, 2019; Whittemore and Knafl, 2005).

### Analysis

The qualitative data analysis software NVivo 14 was used to support the analysis, thereby increasing transparency of the analytical process. Braun and Clarke's (2019) six-stage reflexive thematic analysis framework served as the basis for the analysis. Reflexive thematic analysis involves the organization of data through coding and the distillation of recurring or significant ideas and concepts. A thematic map was generated and evaluated by the review team.

## Results

The results of this review included 15 articles (see Appendix 2). The research reported by the included articles was conducted in Sweden (*n* = 4), Australia (*n* = 2), Canada (*n* = 2), England (*n* = 2), Brazil (*n* = 1), USA (*n* = 1), Norway (*n* = 1), Switzerland (*n* = 1) and the Netherlands (*n* = 1). The limited number of articles examining employee participation in the context of healthcare's digital transformation highlights a significant research gap, which stands as one of the main findings of this review.

**Figure 2:**
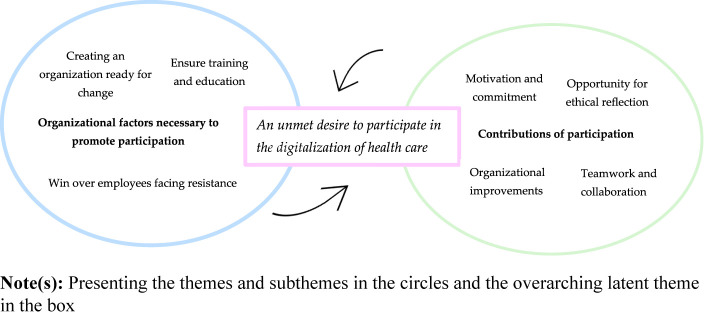
Thematic map of the thematic analysis

The included articles did not all exhibit a primary focus on employee participation; however, they all explored health professionals' perspectives on the digital transformation of healthcare or the implementation of new welfare technologies. The themes revealed by this review are presented below in the thematic map (Figure 2), they involve the organizational factors necessary to promote participation, the contributions of such participation and the imminently latent theme of an unmet desire to participate in the digitalization of healthcare.

### The organizational factors necessary for promoting participation

To promote employee participation, the following themes were identified as important, answering the first research question; what organizational factors are needed for enabling employee participation in the implementation of new technology within healthcare?

#### Knowledge and training

Knowledge and training were identified as important for the successful implementation of health technologies in general (Badawy *et al.*, 2022; Curtis and Brooks, 2020; Frisinger and Papachristou, 2023; Golz *et al.*, 2022; Singh *et al.*, 2022; Wong *et al.*, 2023), and for the achievement of participation of health professionals in the digital transformation (Cuesta *et al.*, 2020; dos Santos and Grativol Aguiar Dias de Oliveira, 2016). Participation of health professionals throughout the implementation process implies that knowledge about both implementation and health technologies in general is important rather than merely information and training regarding the technology to be implemented.

#### Winning over employees who resist change

Hindering characteristics of health professionals included negative attitudes, a lack of inspiration and unwillingness to collaborate (Frisinger and Papachristou, 2023; Golz *et al.*, 2022; Mullender *et al.*, 2022; Nilsen *et al.*, 2019; Wong *et al.*, 2023), furthermore a possible explanation for this negativity was described as involving mandatory changes and many simultaneous changes as well as digital poverty (Mullender *et al.*, 2022; Wilson *et al.*, 2023). To foster participation in healthcare organizations, it is crucial to engage employees by highlighting the benefits of embracing new technologies—a transformation that itself is fueled by active participation.

#### Creating an organization ready for change

The essential organizational factors for promoting employee participation in the implementation of new technologies were identified as the necessary allocation of time and resources (Cuesta *et al.*, 2020; Mullender *et al.*, 2022; Wutzke *et al.*, 2016). Another theme identified focused on the definition of clear roles and responsibilities by managers (Mullender *et al.*, 2022). Other obstacles that were identified included the fact that health organizations are large and therefore slow in changing practices because decisions in these organizations takes time (Mullender *et al.*, 2022). The facilities and environments also could hinder the implementation of new technology as well as pose difficulties to the changing of routines (Mullender *et al.*, 2022). Another barrier to the implementation of new technologies was the use of different words by different professional roles within the organization, which could lead to misunderstandings during the implementation process (Mullender *et al.*, 2022).

### Contributions of participation

The following themes answer the second research question; what employee participation contributes with to technology implementation.

#### Perceived organizational improvements

Health professionals experienced the implementation of new technologies as important when the new technology was expected to result in improvements in the quality of care (Badawy *et al.*, 2022; Bail *et al.*, 2023; Curtis and Brooks, 2020; Dining Zuber and Moody, 2018; Varsi *et al.*, 2023; Wong *et al.*, 2023). The involvement of health professionals in the implementation process seemed to contribute to the implementation of technology that fits the needs of the organization and professionals and technology that could facilitate person-centered care (Bail *et al.*, 2023; Curtis and Brooks, 2020). The participation of employees in the implementation of new technology also ensures that patient safety is maintained (Bail *et al.*, 2023).

#### Motivation and commitment

The participation requires health professionals to be prepared for the new technology from early stages of implementation (Bail *et al.*, 2023). This sense of organizational readiness could be developed by promoting a sense of urgency and fostering a proactive orientation towards change among health professionals (Dining Zuber and Moody, 2018; Mullender *et al.*, 2022; Wutzke *et al.*, 2016). Such a mutual preunderstanding must be holistic, including awareness of the underlying problem that prompts the need for a technical solution as well as insights into how and why the technology can enhance the work processes and the quality of care (Wutzke *et al.*, 2016). In addition to understanding the functioning of the technological solution, later in the process health professionals require advanced training in the utilization of the technology (dos Santos and Grativol Aguiar Dias de Oliveira, 2016).

This preparatory knowledge and training for health professionals may be promoted through clear definitions of the problem statements, the establishment of visible plans prior to implementation (Wutzke *et al.*, 2016), and a collective readiness for change and the adoption of new technology among all staff members (Bail *et al.*, 2023). By fostering the motivation for change and technology implementation (Frisinger and Papachristou, 2023), health professionals gain the ability to innovate, experiment with novel solutions (Dining Zuber and Moody, 2018), and collaboratively address challenges (Dining Zuber and Moody, 2018; dos Santos and Grativol Aguiar Dias de Oliveira, 2016; Frisinger and Papachristou, 2023).

Employee participation is a process that requires more time than traditional top-down implementation (Singh *et al.*, 2022). During this slow process it is necessary to persistently inform professionals about the process to ensure that employee participation are constantly maintained (Dining Zuber and Moody, 2018). As a reward for this slow and time consuming process the organization can motivate employees to feel a sense of ownership and pride, which facilitate future implementation of technology (Frisinger and Papachristou, 2023; Mullender *et al.*, 2022).

#### Opportunity for ethical reflection

Engaging health professionals throughout the digitalization process, ranging from initiation to completion, facilitates a thorough ethical discourse (Cuesta *et al.*, 2020). Such a discourse is crucial, especially given the proven potential for new technologies to introduce novel ethical considerations that have not been previously taken into account (Wilson *et al.*, 2023).

The ethical challenges associated with emerging technologies are diverse and often unpredictable. However, by engaging health professionals in the planning stages of new implementations, an important ethical dialog might be fostered. This collaborative approach incorporates the perspectives of various health professionals, thus enhancing the likelihood that the ethical dilemmas that may arise during and after implementation will be anticipated (Bail *et al.*, 2023; Cuesta *et al.*, 2020; Curtis and Brooks, 2020; Wilson *et al.*, 2023).

#### Teamwork and collaboration

Another theme focuses on the phenomena of teamwork and collaboration which may both arise due to participation in the implementation of new technology and serve as a perquisites for the participation of health professionals in the implementation of new technology (Cuesta *et al.*, 2020; Frisinger and Papachristou, 2023; Mullender *et al.*, 2022). If a safe culture based on teamwork, were everyone is able to express their ideas is not established then employee participation in the implementation of new health technologies may be hindered and become unachievable (Dining Zuber and Moody, 2018; Mullender *et al.*, 2022; Wong *et al.*, 2023).

## Discussion

The purpose of this literature review was to identify essential themes in the literature on health professionals’ views regarding participation in the digital transformation of healthcare. A recurring theme in the literature pertained to the consensus exhibited by health professionals regarding the paramount significance of employee participation in the implementation of new technologies. Although this literature review highlighting the significance of participation, it also emphasizes the fact that the actual involvement of health professionals in the digital transformation has received insufficient exploration and research. The limited number of articles examining employee participation in the context of healthcare's digital transformation with qualitative methods highlights a significant research gap and affects the overall trustworthiness of the findings of this qualitative review. The ways in which employee participation can be achieved and its true contributions to healthcare digital transformation remain underexplored.

To ensure successful employee participation, time must be invested prior to the implementation process. Health professionals should be encouraged to comprehend the benefits of the new technology and to believe that the technology has the potential to improve care quality, otherwise the implementation may be challenging and prolonged, and it may even fail entirely. Theories drawn from the field of QM have highlighted the importance of dedicating ample time to the planning phase of the change processes (Reed and Card, 2016). The planning phase is often skipped in favor of taking quick action in a health care culture that prioritizes “doing” (Muthukrishnan *et al.*, 2022; Rao *et al.*, 2017; Rodrigue *et al.*, 2013).

Previous research and theories from QM have concluded that employee participation is important, and this review highlights its significance in the context of implementation of new technology in healthcare. Employee participation contributes to the possibility of feeling ownership and pride of one’s work, which is expressed by founders of the quality movement like Deming, who promoted the need for management to ensure employees have the opportunity to feel pride of workmanship (Deming, 2000). However, the absence of studies describing the phenomena of employee participation, alongside the core latent theme identified in this review suggests that employee participation in digital transformation is lacking and must be strengthened. The question of why this goal is challenging to achieve remains unanswered. Some researchers have argued that this situation is caused by the entrenched influence of the new public management culture, which is characterized by an emphasis on economics, time constraints, staffing shortages, and insufficient slack time (Hasselbladh *et al.*, 2008) Research on the ways in which healthcare organizations can change the prevailing culture and foster employee participation is necessary. One solution to this problem may be to integrate QM into improvement efforts, as QM theories strongly advocate for employee participation (Bergman *et al.*, 2022).

Time and resources appear to represent primary obstacles to the task of promoting employee participation. This finding is in line with prior research, which has focused on context of limited resources in healthcare organizations (Davies *et al.*, 2014; Holmér *et al.*, 2023; Lynch *et al.*, 2010; Sommerbakk *et al.*, 2016). This literature review indicates that the organization gains more motivated and committed employees, as well as an improved context of teamwork and opportunities for ethical reflection when promoting participation. These values cannot be measured economically. The aim of digitalization is to gradually reduce the redundant tasks faced by health professionals and guarantee the delivery of high-quality care to a larger number of patients, especially in situations featuring a shortage of skilled health care workers. However, the pursuit of the implementation of a new technology without ensuring a successful integration process becomes paradoxical. This oversight not only prolongs the adoption period and hampers the effective utilization of the technology but also imposes additional stress and a higher workload on an already overburdened workforce.

*Limitations:* This review may have several limitations. It may be subject to language bias due to the inclusion solely of articles that were written in English. Additionally, it is possible that relevant articles were missed, as the searches were conducted by a single researcher; however, efforts were made to mitigate this possibility by implementing a rigorous search strategy and seeking assistance from university librarians.

### Conclusions

The latent analysis revealed a main latent theme pertaining to an unmet desire of health professionals to participate in the digital transformation of healthcare. This sentiment is understandable given that health professionals consistently emphasize the significance of active participation, often citing cases in which implementation projects faltered due to insufficient opportunities for participation. This claim is supported by a quantitative study that concluded that health professionals must be provided with more opportunities to actively participate in the implementation of new technology than are currently available (Baudin *et al.*, 2020). The experiences of health professionals outlined in the included studies consistently highlighted employee participation as a crucial determinant of success. This finding is in line with prior research indicating that the participation of health professionals in improvement work represents a critical success factor in this context (Blake *et al.*, 2006; Keroack *et al.*, 2007). The participation of frontline staff seems to be the factor that truly matters with regard to improvements in quality (Irwin *et al.*, 2013; Sinkowitz-Cochran *et al.*, 2012; Tavernier *et al.*, 2018).

The articles included in this review are primarily from Western countries, particularly Northern Europe, the United States, and Australia—regions recognized as leaders in the digital transformation of healthcare. According to Majcherek *et al.* (2024), Northern European countries are notably ahead in digital healthcare innovation compared to the rest of Europe. Consequently, the findings of this qualitative review may be most relevant to Northern Europe and other highly digitalized nations at the forefront of healthcare digitalization.

### Recommendations for healthcare managers to overcome barriers in digital transformation

(1) *Maximize employee participation with QM principles:* Although employee participation can be time-consuming and challenging, actively encouraging participation is crucial. Doing so will strengthen the implementation, ensuring the technology is effectively used and delivers value to both patients and staff. Principles and techniques from Quality Management can help achieve employee participation.
(2) *Create teamwork through inclusion:* Participation promotes stronger teamwork. Involving various health professionals with different expertise and perspectives will further enhance collaboration and the overall success of the implementation.
(3) *Prioritize ethical reflection with evidence from research:* Ethical considerations regarding new technologies must be prioritized at every stage—before, during, and after implementation—to ensure responsible and evidence-based integration into healthcare practices.

## Directions for future research

This review highlights the necessity of future research on the broader phenomenon of healthcare digital transformation and the role of employee participation in this process. More studies are needed to investigate how employee participation in digital transformation can be promoted by healthcare organizations, and to answer this question studies from various perspectives, including both health professionals and the managers of healthcare organizations, are needed. The research gap identified in this literature review is further supported by other reviews in the field of welfare technology implementation, thereby highlighting the status of this context as an underexplored area (Borg *et al.*, 2022).

## Appendix 1

The list of search terms for the qualitative systematic literature review:

Table A1

**Table A1:** Presentation of the search terms

Field	Operator	Keywords
”Digital Transformation”	OR	Technology OR Digital, Digitalization OR digitalization OR implement* OR innovation OR improvement OR “welfare technology” OR digitalize OR digitalize OR digitiz* OR digitis* OR “health innovation” OR “diffusion of innovation” OR “innovation diffusion” OR “disruptive technology”
	AND	
“Health Professionals”	OR	Nurse OR “nursing staff” OR “nursing personnel” OR “healthcare staff” OR “healthcare personnel” OR “healthcare worker” OR “Health worker” OR “healthcare professional” OR “health care practitioner” OR “health-care worker” OR “health-care personnel” OR “health-care professional” OR “health-care practitioner”
	AND	
Participation	OR	Satisfaction OR engagement OR motivat* OR involvement OR empower* OR participat* OR autonom* OR creativ* OR commitment OR well-being

## Appendix 2

Qualitative primary articles included in the literature review:

Table A2

**Table A2:** Presentation of the included articles in the review

Authors	Location	Findings related to participation in the implementation of new technology
Badawy *et al*	Norway	Knowledge and training were identified as important for the successful implementation of health technologies. Health professionals experienced the implementation of new technologies as important when the new technology was expected to result in improvements in the quality of the care provided
Bail *et al*	Australia	The involvement of health professionals in the implementation process contributed to the implementation of technology that fits the needs of the organization and could facilitate person-centered care. The participation also ensures that patient safety is maintained. Participation requires health professionals to be prepared for the new technology from early stages of implementation
Cuesta *et al*	Sweden	Engaging health professionals throughout the digitalization process, ranging from initiation to completion, facilitates a thorough ethical discourse. A collaborative approach incorporates the perspectives of various health professionals, thus enhancing the likelihood that the ethical dilemmas that may arise during and after implementation will be anticipated. Teamwork and collaboration may both arise due to participation in the implementation of new technology and serve as a perquisite for the participation of health professionals in the implementation of new technology
Curtis and Brooks	England	The ethical challenges associated with emerging technologies are diverse and often unpredictable, by engaging health professionals in the initial planning stages of new implementations, health care organizations may foster an important ethical dialog
Dining Zuber and Moody	USA	By fostering the motivation for change and technology implementation, health professionals gain the ability to innovate, experiment with novel solutions, and collaboratively address challenges. The sense of organizational readiness could be developed by promoting a sense of urgency and fostering a proactive orientation towards change among health professionals
dos Santos *et al*	Brazil	In addition to understanding the functioning of the technological solution, later in the process health professionals require advanced training in the utilization of the technology in question. By fostering the motivation for change and technology implementation, health professionals gain the ability to innovate, experiment with novel solutions, and collaboratively address challenges
Frisinger and Papachristou	Sweden	By fostering the motivation for change and technology implementation, health professionals gain the ability to innovate, experiment with novel solutions, and collaboratively address challenges. As a reward for this slow and time-consuming process the organization can motivate employees to feel a sense of ownership and pride, which seems to facilitate future implementation of technology and creates readiness for change within the organization
Golz *et al*	Switzerland	Knowledge and training were identified as important both for the successful implementation of health technologies in general. Hindering characteristics of health professionals included negative attitudes, a lack of inspiration and the lack of unwillingness to collaborate
Mullender *et al*	Netherlands	The essential organizational factors for promoting employee participation in the implementation of new technologies were identified as the necessary allocation of time and resources. Another theme identified as necessary focused on the definition of clear roles and responsibilities by managers. Other obstacles that were identified included the fact that health organizations tend to be large and therefore slow with regard to changing practices because decisions in these organizations tend to take time
Nilsen *et al*	Sweden	Hindering characteristics of health professionals included negative attitudes, a lack of inspiration and the lack of unwillingness to collaborate
Singh *et al*	Canada	Knowledge and training were identified as important both for the successful implementation of health technologies. Employee participation is a time-consuming process that requires more time than a traditional top-down implementation process
Varsi *et al*	Norway	Health professionals experienced the implementation of new technologies as important when the new technology in question was expected to result in improvements in the quality of the care provided
Wong *et al*	Canada	If a safe culture based on teamwork, in which context everyone can express their ideas is not established then employee participation in the implementation of new health technologies may be hindered and become unachievable
Wutzke *et al*	Australia	A mutual preunderstanding must be holistic, including awareness of the underlying problem that prompts the need for a technical solution as well as insights into how and why the technology in question can enhance the work processes and the quality of care. The preparatory knowledge and training for health care professionals may be promoted to some extent through clear definitions of the problem statements, the establishment of visible plans prior to implementation
Wilson *et al*	England	Engaging health professionals throughout the digitalization process, ranging from initiation to completion, facilitates a thorough ethical discourse. Such a discourse is crucial, especially given the proven potential for new technologies to introduce novel ethical considerations that have not been previously considered
